# The Role of FADS1/2 Polymorphisms on Cardiometabolic Markers and Fatty Acid Profiles in Young Adults Consuming Fish Oil Supplements

**DOI:** 10.3390/nu6062290

**Published:** 2014-06-16

**Authors:** Kaitlin Roke, David M. Mutch

**Affiliations:** Department of Human Health and Nutritional Sciences, University of Guelph, Guelph, Ontario, N1G2W1, Canada; E-Mail: kroke@uoguelph.ca

**Keywords:** eicosapentaenoic acid (EPA), docosahexaenoic acid (DHA), omega-3 (*n*-3) fatty acids, single nucleotide polymorphisms (SNPs), fatty acid desaturase 1 and 2 (FADS1/2), serum, red blood cells (RBC), triglycerides (TAG), glucose

## Abstract

Eicosapentaenoic acid (EPA) and docosahexaenoic acid (DHA) are omega-3 (*n*-3) fatty acids (FAs) known to influence cardiometabolic markers of health. Evidence suggests that single nucleotide polymorphisms (SNPs) in the fatty acid desaturase 1 and 2 (FADS1/2) gene cluster may influence an individual’s response to *n*-3 FAs. This study examined the impact of a moderate daily dose of EPA and DHA fish oil supplements on cardiometabolic markers, FA levels in serum and red blood cells (RBC), and whether these endpoints were influenced by SNPs in FADS1/2. Young adults consumed fish oil supplements (1.8 g total EPA/DHA per day) for 12 weeks followed by an 8-week washout period. Serum and RBC FA profiles were analyzed every two weeks by gas chromatography. Two SNPs were genotyped: rs174537 in FADS1 and rs174576 in FADS2. Participants had significantly reduced levels of blood triglycerides (−13%) and glucose (–11%) by week 12; however, these benefits were lost during the washout period. EPA and DHA levels increased significantly in serum (+250% and +51%, respectively) and RBCs (+132% and +18%, respectively) within the first two weeks of supplementation and remained elevated throughout the 12-week period. EPA and DHA levels in RBCs only (not serum) remained significantly elevated (+37% and +24%, respectively) after the washout period. Minor allele carriers for both SNPs experienced greater increases in RBC EPA levels during supplementation; suggesting that genetic variation at this locus can influence an individual’s response to fish oil supplements.

## 1. Introduction

The Western diet is characterized as being rich in omega-6 (*n*-6) polyunsaturated fatty acids (PUFA) and poor in omega-3 (*n*-3) PUFA, specifically eicosapentaenoic acid (EPA) and docosahexaenoic acid (DHA) [1,2]. Individuals can increase their EPA and DHA levels by consuming fatty fish, *n*-3 fortified foods, or dietary supplements such as fish oil, algal oil, or krill oil [3,4]. It is well documented that consuming fish oil leads to reductions in cardiometabolic markers including blood triglycerides (TAG) and inflammatory mediators, which may reduce the risk for cardiovascular and metabolic diseases [5,6,7]. Furthermore, individuals with higher baseline fasting TAG levels experience proportionally greater decreases in TAG with fish oil supplementation [8,9]. In contrast, changes in other cardiometabolic markers such as cholesterol, glucose, and insulin, have shown conflicting results with fish oil supplementation [8,10]. Reasons for these discrepancies include differences in study populations with respect to age, sex, and disease status, as well as differences in study design such as dose and length of supplementation [8,11,12,13]. Young healthy adults are one age group in which the changes in cardiometabolic markers with fish oil supplementation remains poorly characterized. 

Changes in fatty acid (FA) intake, be it from foods or dietary supplements, are reflected in blood and can be quantitatively measured in distinct blood fractions such as serum, plasma, and red blood cells (RBC) [6,14]. It has previously been reported that FA profiles in different blood fractions are altered in response to fish oil supplements providing between 2.2 and 4.8 g EPA/DHA per day [15,16,17,18]. However, less is known regarding the extent by which FA profiles change in various blood fractions during and after the consumption of fish oil supplements providing a moderate dose of 1.8 g EPA/DHA per day. Understanding the dynamic changes in FA profiles in various blood fractions will help build knowledge regarding the length of time fish oil supplements are required to be taken in order to achieve and maintain increases or decreases in specific FAs. The changes in these FA profiles may also contribute towards alterations in cardiometabolic markers.

In addition to the relationships between FAs and cardiometabolic health, there is also evidence demonstrating that variation in the fatty acid desaturase 1 and 2 (FADS1/2) gene cluster can influence cardiometabolic markers. For example, single nucleotide polymorphisms (SNPs) in the FADS1/2 gene cluster have been associated with diseases such as cardiovascular disease and type 2 diabetes [19,20,21,22,23]. However, less research has focused on these SNPs and their effects on individual cardiometabolic markers. Previous research in our lab [24] and elsewhere [25] have shown that SNPs in the FADS1/2 gene cluster are related to changes in circulating hsCRP levels; a marker of whole body inflammation. In addition, Cormier *et al.* [26] have recently examined the influence of FADS1/2 SNPs on blood TAG levels. Evidence from studies supplementing with flaxseed oil or encapsulated EPA/DHA suggests that genetic variation in FADS1/2 can affect how a person responds to *n*-3 FA supplements [27,28]. Further, these studies in middle-aged adults showed that minor allele carriers for SNPs in the FADS1/2 gene cluster have lower blood EPA levels before supplementation [27,28]. Therefore, it is important to see if these findings are also seen in young adults consuming fish oil supplements.

The objectives of the present study were to assess the impact of fish oil supplementation, providing 1.8 g EPA/DHA per day, in young adults (18–25 years of age) using several approaches. First, we examined whether fish oil supplementation could lead to reductions in cardiometabolic markers. Second, we measured changes in FA profiles in both serum and RBC fractions during a 12-week fish oil supplementation period, followed by an 8-week washout period. Last, we examined to what extent changes in cardiometabolic markers and blood FA profiles were influenced by common SNPs in the FADS1/2 gene cluster. Overall, we anticipate that the findings of this study will help generate further support that knowledge of an individual’s FADS1/2 genotype may help guide the development of personalized strategies using *n*-3 FA supplements to improve health and prevent disease.

## 2. Methods

### 2.1. Participant Characteristics and Omega-3 Supplementation

Young male adults (*n* = 12) between 18 and 25 years of age were recruited from the University of Guelph through study posters. An *n*-3 diet survey was used during the initial screening to ensure that participants had not consumed fish oil supplements in the past 12 weeks and/or were not frequent consumers of foods rich or fortified with *n*-3 FAs. Participants were instructed to maintain regular exercise and dietary habits throughout the entire study. The study consisted of a 12-week fish oil supplementation followed by an 8-week washout. During the supplementation period, participants consumed three Clearwater Omega-3 capsules per day (kindly provided by Ocean Nutrition Canada Ltd., Nova Scotia, Canada) providing a total of 1200 mg EPA and 600 mg DHA per day. Participants were instructed to consume fish oil capsules with meals, as this was previously shown to improve absorption [29]. Compliance was assessed by analyzing EPA and DHA levels in both serum and RBC fractions at baseline and throughout the study. Anthropometric measurements (age, weight, height, and calculated BMI) were taken at baseline and at the end of the washout period. This study (Clinicaltrials. gov. identifier NCT02042274) was approved by the University of Guelph Research Ethics Board (REB #12JL006).

### 2.2. Analysis of Cardiometabolic Markers

**Figure 1:**
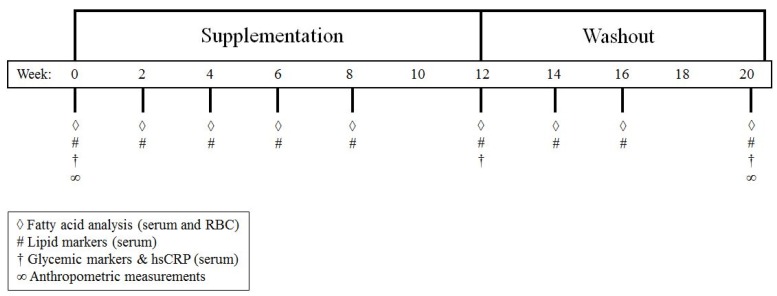
Study timeline and experimental design. The supplementation period lasted for 12 weeks and the washout period for 8 weeks (total duration of 20 weeks). Symbols indicate which measurements were taken at each time-point.

Blood samples were collected by venipuncture during the 20-week study period at baseline and weeks 2, 4, 6, 8, 12, 14, 16 and 20 (study timeline Figure 1). Lipid markers including TAG, total cholesterol, LDL-cholesterol (LDL-c), HDL-cholesterol (HDL-c), and the cholesterol/HDL-c ratio were measured at each time point. Glycemic parameters (glucose, insulin, and glycated hemoglobin (HbA1c)) and an inflammatory marker (high sensitivity C-reactive protein (hsCRP)) were measured at baseline, week 12, and week 20. All blood samples used for the analysis of these cardiometabolic markers were sent to Lifelabs immediately after collection and analyzed using standard laboratory procedures.

### 2.3. Fatty Acid Analysis

Blood samples were collected at each study visit, centrifuged to separate serum and RBCs, and then aliquoted and stored at −80 °C until analysis. The FA profile was measured by gas chromatography as previously described [30]. FA peaks were identified by comparison to retention times of FA methyl ester standards. Individual FAs are indicated as a percentage (%) of total FAs. Specific FAs including EPA, DHA, arachidonic acid (AA), docosapentaenoic acid (DPA), α-linolenic acid (ALA), γ-linolenic acid (GLA), and linoleic acid (LA) are reported. 

### 2.4. Analysis of Variants in the FADS1/2 Gene Cluster

DNA was extracted using the Qiagen PAXgene blood DNA kit, according to manufacturer instructions (Qiagen, Toronto, Ontario, Canada). DNA quality was visually confirmed on a 1% agarose gel. Two SNPS were genotyped: rs174537 in FADS1 (Chr11:61785208, MAF = 0.33), and rs174576 in FADS2 (Chr11:61836038, MAF = 0.39) [31]. These SNPs were selected for the current analysis because several reports (both by us and others) have showed them to be reproducibly associated with blood FA levels and cardiometabolic markers [20,24,30,32,33]. Detection of the rs174537 SNP in FADS1 and the rs174576 SNP in FADS2 was carried out using validated TaqMan genotyping assays (Assay ID C_2269026_10_37 and C_2575520_10_76, respectively; Life Technologies, CA, USA) and each sample was analyzed in triplicate to ensure genotyping accuracy. Amplification was conducted on a Bio-Rad CFX96 Real-Time PCR Detection System (Bio-Rad, CA, USA) using an amplification protocol of 95 °C for 10 minutes followed by 40 cycles, which included denaturing for 15 s at 92 °C and annealing/extending for 1 min at 60 °C. Allelic discrimination was performed using CFX96 software to distinguish between different genotypes: major allele homozygotes (GG for rs174537; CC for rs174576), heterozygotes, and minor allele homozygotes (TT for rs174537; AA for rs174576). Linkage disequilibrium (LD) was determined *post-hoc* to examine the two SNPs used in our study, as well as compared to other SNPs reported in the literature. LD was determined with SNAP (SNP annotation and proxy search [34]) using the CEU population as the reference group, with data generated as per Pettersson *et al.* [35].

### 2.5. Statistical Analysis

Changes in serum and RBC FAs were determined by comparing each time point to baseline values using a non-parametric Wilcoxon paired *t*-test. All genotype analyses were conducted using a dominant model. FA differences according to genotype were assessed at baseline, week 12, and week 20 using a non-parametric Mann-Whitney *U*-test. A genotype × time interaction was used to examine the impact of changes in specific FAs throughout the study period. GraphPad Prism 5 (GraphPad Software, Inc., CA, USA) and JMP Genomics software V5 (SAS Institute, Cary, NC, USA) were used for all analyses. A *p* < 0.05 was considered statistically significant.

## 3. Results

### 3.1. Fish Oil Supplementation and Cardiometabolic Markers

All 12 participants completed the study. Anthropometric data and cardiometabolic markers are indicated in Table 1. The average age of participants was 21.8 ± 1.1 years at baseline. Participants showed a significant decrease in fasted TAG (−13%, *p* = 0.03) and glucose (−11%, *p* = 0.03) levels by the end of the 12-week supplementation period. These changes were not maintained during the washout period, where both fasted TAG and glucose levels rebounded back to baseline values (Table 1). No significant changes were detected for total cholesterol, LDL-c, HDL-c, the cholesterol/HDL ratio, fasting insulin, HbA1c, or hsCRP during the 20-week study period (Table 1).

**Table 1:** Participant characteristics. Average anthropometric and clinical measures are listed at baseline, week 12, and week 20 for study participants (*n* = 12). Values are indicated as mean ± SD. * Indicates *p* < 0.05 compared to baseline. ND, not determined.

Parameter	Baseline	Week 12	Week 20
BMI (kg/m^2^)	25.7 ± 4.0	ND	25.8 ± 4.2
Triglyceride (mmol/L)	0.9 ± 0.3	0.8 ± 0.3 *	0.9 ± 0.5
Total cholesterol (mmol/L)	4.0 ± 0.5	4.2 ± 0.7	4.0 ± 0.5
LDL-c (mmol/L)	2.4 ± 0.5	2.5 ± 0.5	2.4 ± 0.4
HDL-c (mmol/L)	1.3 ± 0.2	1.3 ± 0.3	1.3 ± 0.2
Cholesterol: HDL-c	3.2 ± 0.6	3.3 ± 0.5	3.2 ± 0.5
Glucose (mmol/L)	4.4 ± 1.4	3.9 ± 1.3 *	4.2 ± 1.4
Insulin (ρmol/L)	54.5 ± 22.1	46.8 ± 26.2	57.6 ± 28.5
HbA1c (mmol/L)	0.05 ± 0.002	0.05 ± 0.002	0.05 ± 0.002
hsCRP (mg/L)	1.4 ± 1.2	1.4 ± 1.3	1.2 ± 1.0

### 3.2. Fatty Acid Profiles in Serum and RBCs

**Figure 2:**
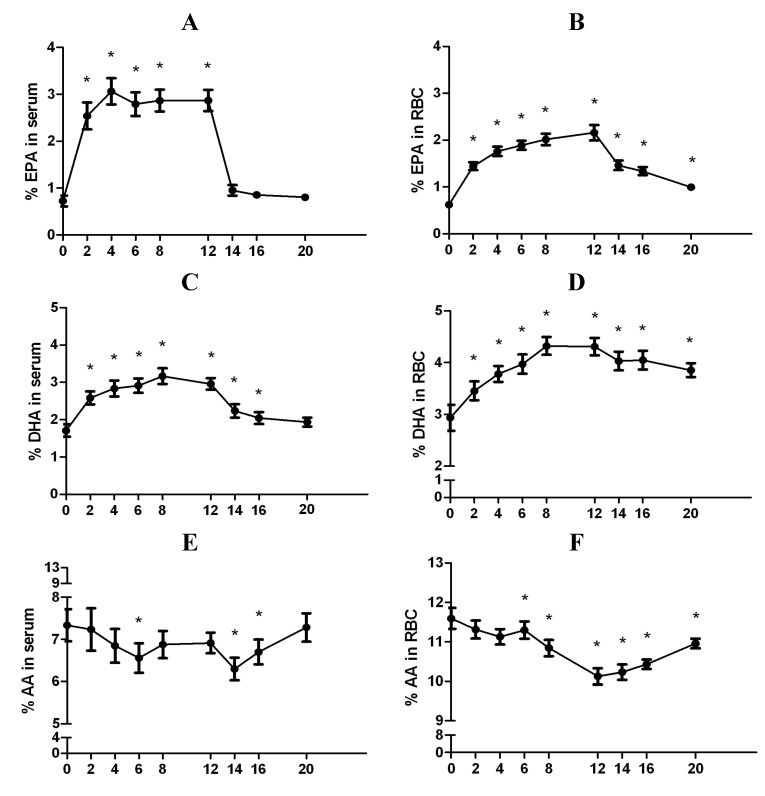
Levels of eicosapentaenoic acid (EPA), docosahexaenoic acid (DHA), and arachidonic acid (AA) in serum and RBCs. Relative % EPA in (**A**) serum and (**B**) RBCs. Relative % DHA in (**C**) serum and (**D**) RBCs. Relative % AA in (**E**) serum and (**F**) RBCs. FAs are represented as relative % of total FA values. Numbers on the *x*-axis represent time in weeks, where baseline is indicated by 0. * Indicates *p* < 0.05 compared to baseline (0).

Within two weeks of commencing fish oil supplementation, EPA and DHA levels were significantly increased in both serum (+250%, *p* = 1.0 × 10^−3^ and +51%, *p* = 5.0 × 10^−4^, respectively) and RBC (+132%, *p* = 1.0 × 10^−3^ and +18%, *p* = 2.0 × 10^−3^, respectively) fractions (Figure 2A–D). The levels of EPA and DHA remained significantly elevated throughout the supplementation period. During the washout period, serum EPA levels returned to baseline values within two weeks of supplementation ending. In comparison, serum DHA levels remained significantly elevated within two weeks after supplementation had ceased, but returned to baseline levels by the end of the 8-week washout period. In RBCs, EPA and DHA levels also decreased during the washout period; however, they remained significantly elevated at the end of the 8-week washout period compared to baseline (+37%, *p* = 1.0 × 10^−3^ and +24%, *p* = 4.9 × 10^−3^, respectively). Arachidonic acid (AA) levels decreased in serum during supplementation; however, there was considerable inter-individual variability resulting in sporadic significance during the 20 week study (Figure 2E). In contrast, AA levels in RBCs were significantly decreased by the sixth week in the supplementation period, and were significantly reduced (−13%, *p* = 1.0 × 10^−3^) compared to baseline, at week 12 (Figure 2F). Similar to EPA and DHA, AA levels in RBCs remained significantly altered by the end of the washout period in comparison to baseline, with a significant decrease (−6%, *p* = 0.02).

While EPA, DHA, and AA are the primary FAs that we expected to see changed with fish oil supplementation, we also examined other FAs detected by gas chromatography. By the end of the supplementation period, we noted significant decreases in serum levels of linoleic (LA) and γ-linoleic acids (GLA) (−9%, *p* = 2.4 × 10^−3^ and −34%, *p* = 2.4 × 10^−3^, respectively), and significant increases in docosapentaenoic acid (DPA) (+49%, *p* = 5.0 × 10^−4^) compared to baseline. After the washout period, serum levels of LA, GLA, and DPA had all returned to baseline values. RBC levels of LA decreased (−13%, *p* = 1.5 × 10^−2^), and there were significant increases in RBC DPA levels (+39%, *p* = 1.5 × 10^−2^) at week 12 compared to baseline. While RBC LA levels returned to baseline during the washout period, RBC DPA levels remained significantly increased (+22%, *p* = 4.9 × 10^−3^). We observed no significant changes in the levels of α-linolenic acid (ALA) in either serum or RBCs during the 20-week study. Moreover, no significant changes were detected for any saturated or monounsaturated FAs during the study.

### 3.3. Analysis of Variants in the FADS1/2 Gene Cluster

**Figure 3:**
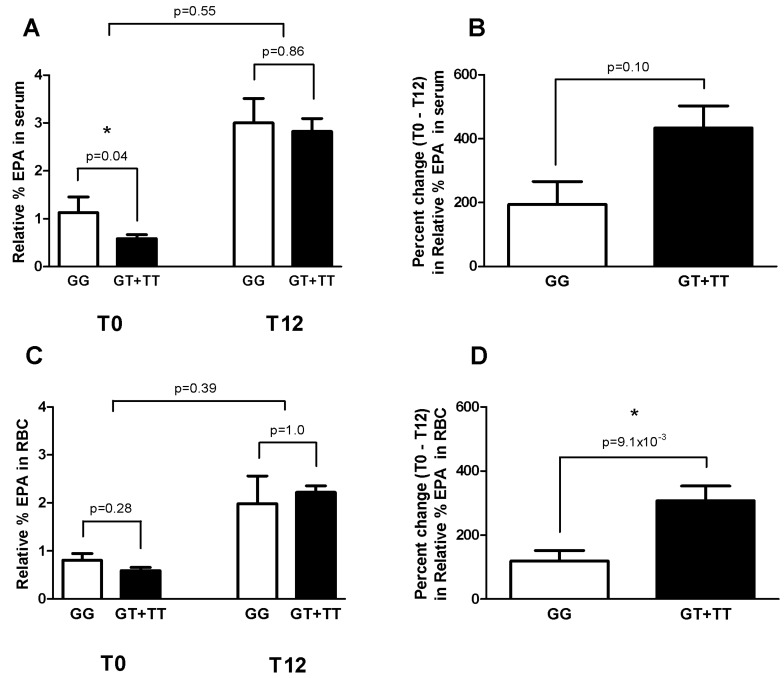
Eicosapentaenoic acid (EPA) levels in major and minor allele carriers of the rs174537 SNP in FADS1. (**A**) Differences in relative % EPA in serum between major and minor allele carriers at both baseline (T0) and week 12 (T12) as well as the interaction between genotype × time. (**B**) Percent changes (*i.e.*, T0 − T12) for serum % EPA in major and minor allele carriers. (**C**) Differences in relative % EPA in RBCs between major and minor allele carriers at both T0 and T12 as well as the interaction between genotype × time. (**D**) Percent change (*i.e.*, T0 − T12) for RBC % EPA in major and minor allele carriers. *p*-Values are listed above each of the comparisons. Major allele carriers (GG) (*n* =3) and minor allele carriers (GT+ TT) (*n* = 9). * Indicates *p* < 0.05.

Genotyping results revealed identical allelic distributions for individual participants for the two SNPs analyzed (rs174537 in FADS1 and rs174576 in FADS2). A *post-hoc* examination of LD revealed that these two SNPs are in perfect LD (1.0); thus indicating that identical allelic distribution was not due to chance. As such, we chose to present findings for the rs174537 SNP only. The influence of these SNPs on cardiometabolic markers and FA levels (EPA, DHA, and AA) was examined at baseline, week 12, and week 20 of the study. There were no significant differences in cardiometabolic markers (specifically TAG or glucose levels) between major (GG; *n* = 3) and minor (GT + TT; *n* = 9) allele carriers at any time point. There was a significant difference in serum EPA levels between major and minor allele carriers at baseline, with minor allele carriers showing lower baseline serum EPA levels (−48%, *p* = 0.04; Figure 3A). A similar trend was observed for EPA levels in RBC, but did not reach statistical significance (−27%, *p* = 0.28; Figure 3C). While EPA levels increased in both major and minor allele carriers after the 12-week supplementation period, we were unable to detect a significant genotype × time interaction in either serum or RBCs (Figure 3A,C). However, when expressing changes in EPA levels as a percent (%) change (between baseline and week 12) (Figure 3B,D), we found that minor allele carriers had a greater increase in RBC EPA levels (*p* = 9.1 × 10^−3^, Figure 3D) during supplementation compared to major allele carriers. Importantly, there was no difference in ALA levels in either serum or RBCs between major and minor allele carriers at any time point; suggesting that changes in ALA intake were not responsible for changes in EPA (data not shown). We observed no significant differences in DHA or AA levels in serum or RBCs at any time point when subjects were stratified by genotype. 

## 4. Discussion

Studies have shown that the increased intake of EPA and DHA from fish oil supplements is linked to reductions in cardiometabolic markers, improvements in overall health, and cardiovascular and metabolic disease prevention [3,5,7,8,36]. It was uncertain if cardiometabolic markers would change in young adults consuming a moderate dose of fish oil supplements (providing 1.8 g EPA/DHA per day) since these individuals already have normal levels of these markers [37,38]. The results of our study demonstrated that young men experienced reductions in cardiometabolic markers, specifically TAG and glucose, with the daily consumption of a moderate dose of fish oil. There were no significant changes in cholesterol parameters after fish oil supplementation, which is consistent with other studies [8,39,40,41,42]. The present study contributes to the field by showing that young healthy individuals also experience reductions in select cardiometabolic markers with a moderate dose of fish oil supplementation in comparison to the doses more commonly reported in the literature (*i.e.*, >3.0 g EPA/DHA per day). However, it is important to note that the reductions in TAG and glucose were quickly lost upon cessation of supplementation; suggesting that improvements in cardiometabolic markers are only maintained with continuous supplementation. Our findings have potential relevance in the context of future disease prevention, as cardiometabolic markers such as TAG are known to increase in an age-dependent manner; thus potentially increasing an individual’s risk for cardiovascular complications [36]. Therefore, supplementing with fish oil in early adulthood could promote a more favorable cardiometabolic marker profile in the longer term. Future longitudinal research is needed in order to determine if the decreases in cardiometabolic markers seen in young adults consuming *n*-3 supplements prevents the development of disease later in life. 

In addition to changes in cardiometabolic markers of health, it has been previously shown that changes in *n*-3 FA intake are reflected in blood FA profiles [14]. For example, increasing EPA and DHA intake causes these FAs to be increased in various blood fractions (serum, plasma, and RBCs) [14,17]. The concentrations of these FAs rise more quickly in plasma and serum in comparison to RBCs, which is expected because RBCs have a turnover rate of approximately 90 days [15,17,39]. As such, RBCs reflect longer-term adaptations in FA intake. We found that EPA levels increased by +250% and DHA increased by +51% in serum within the first two weeks of supplementation compared to baseline. During the washout period, serum EPA and DHA levels decreased back to baseline levels. Specifically, our data showed that serum EPA levels rapidly returned to baseline levels within two weeks of stopping fish oil supplementation, while serum DHA returned to baseline levels only by the end of the washout period. Comparing serum to RBCs, the levels of EPA and DHA in RBCs remained significantly higher by the end of the washout period, most probably due to the 90-day turnover rate of RBCs. Overall, DHA maintained slightly higher levels in both blood fractions during and after supplementation compared to EPA, which is consistent with prior studies using either fish oil supplements providing ~2 g EPA/DHA per day or cod liver oil [16,18,40]. EPA in serum and RBCs increased and decreased more rapidly during the study period than DHA, which also agrees with previous reports studying higher doses of *n*-3 FA supplements (*i.e.*, 2.2–4.8 g EPA/DHA per day) in adults between 21 and 49 years of age [15,16].

While changes in EPA and DHA are expected with fish oil supplementation, it is also interesting to examine if other FAs are changed with supplementation. We found that AA (an *n*-6 FA) levels were reduced slightly in serum and significantly in RBCs during the supplementation period. This aligns with a previous study from our group in which similarly aged men consumed a fish oil supplement providing 3 g EPA/DHA per day for 12 weeks [43]. To expand from this previous work, we have now examined the dynamic changes in AA levels in both serum and RBCs throughout this 12-week supplementation period and 8-week washout period. When examining AA levels throughout the study, we noted that AA levels were more consistently reduced in RBCs compared to serum. In regards to other FAs, we found that serum and RBC DPA levels were significantly increased, and both serum and RBC LA levels were significantly reduced by the end of the fish oil supplementation period. Changes in DPA are likely attributed to the increased amounts of EPA available to be elongated by the fatty acid elongase 2 (ELOVL2) enzyme. Our results are consistent with other studies showing that fish oil supplementation caused increases in serum and RBC DPA [18,43] and a decrease in both serum and RBC LA [18]. The rapid return of serum FAs to initial levels once supplementation ceased highlights the importance of continued supplementation in order to maintain elevated EPA and DHA and reduced AA levels [44,45]. The sustained increases in EPA and DHA levels (particularly in RBCs) during the washout period also demonstrates that care must be taken when designing FA supplementation trials with a cross-over component in order to avoid residual FA changes from the previous supplementation. Importantly, many studies use a washout period of four weeks, which our data suggests would be insufficient to clear changes in FAs such as EPA, DHA, and AA in RBCs [16,18].

It is now recognized that the fatty acid desaturase enzymes from the FADS1/2 gene cluster influence the FA profile in blood fractions and tissues, as well as cardiometabolic markers. Of relevance to the current study, these parameters can also be modified with fish oil supplements [46]. Work by Cormier *et al.* [26] examined the role of several SNPs in the FADS1/2 gene cluster as a potential factor that could influence the changes in TAG levels seen with fish oil supplementation. The authors reported that TAG levels were associated with the rs174546 SNP in FADS1 [26]. Although the two SNPs examined in our study (rs174537 and rs174576) were found to be in perfect LD (1.0) with the rs174546 SNP used by Cormier *et al.* [26] (*post-hoc* LD analysis outlined in Methods), we were unable to replicate this finding; however, this may simply be due to the small size of our study cohort. Nevertheless, our findings agree with the overall conclusions from Cormier *et al.* [26], as both studies found that fish oil supplementation resulted in a significant decrease in TAG levels that was independent of variation in the FADS1/2 gene cluster [26]. In our study, we were unable to show that glucose levels and other cardiometabolic markers were significantly different when stratifying subjects by genotype before, after, or in response to fish oil supplementation. When examining FA levels, we found that minor allele carriers had significantly lower serum EPA levels at baseline (*p* = 0.04), thus agreeing with recent findings reported by Gillingham *et al.* [27] and Al-Hilal *et al.* [28] who used flaxseed oil and encapsulated EPA and DHA, respectively, as sources of *n*-3 supplementation. Due to our small sample size, we were unable to show statistically significant genotype × time interactions in serum or RBCs (Figure 3A,C). To mitigate inter-individual variability in EPA levels, we also reported EPA levels as percent changes (*i.e.*, T0 − T12) (Figure 3B,D). This approach revealed that minor allele carriers experienced a significantly greater percent change increase in RBC EPA during *n*-3 FA supplementation compared to major allele carriers, which agrees with a previous report by Gillingham *et al.* [27]. In contrast, we did not observe a significant genotype effect regarding DHA levels in either serum or RBCs. This may be due to a lower amount of DHA (compared to EPA) in our fish oil supplement. As such, providing higher amounts of DHA in supplements may reveal a genotype effect similar to that seen with EPA. Overall, the results from our study align well with those previously reported by Cormier *et al.* [26], Gillingham *et al.* [27] and Al-Hilal *et al.* [28]. As such, we anticipate that these findings will help to further demonstrate the potential role of variants in the FADS1/2 gene cluster as mediators of the changes in FA profiles and cardiometabolic markers seen with *n*-3 supplementation. 

Based on the growing body of literature highlighting the relationship between variants in the FADS1/2 gene cluster and FA levels, we believe this locus has potential to be a nutrigenomics target that can be used to help guide the use of *n*-3 supplements [36]. As reported, minor allele carriers for SNPs in the FADS1/2 gene cluster typically have lower baseline serum and plasma EPA and AA levels [24,28,30,33,47]. Additionally, individuals with lower baseline EPA levels appear to experience greater increases in these FAs with *n*-3 supplementation [18,48]. We believe our study contributes to the growing body of evidence suggesting that genotyping the FADS1 and FADS2 genes, as well as measuring baseline blood EPA levels, may enable health care professionals to use this information to personalize nutritional recommendations in which *n*-3 supplements are considered.

We acknowledge certain limitations with our study. Firstly, young males were recruited in order to avoid sex-specific differences in FA metabolism; as females experience changes in levels of *n*-3 FAs throughout the menstrual cycle and generally have a higher level of DHA in blood than males [13]. In future studies, both males and females should be considered. In addition, as this population was considered relatively healthy, it is possible that some of the cardiometabolic markers may be modified to a greater extent in older individuals or those with metabolic complications such as hyperlipidemia [8,9,11,12]. We also acknowledge that we have a small sample size; however, we are confident in our data because of the high level of agreement with previous independent studies reporting similar findings in large cohorts. As such, we feel that our work lends additional support regarding the biological relevance of the relationship between *n*-3 FA supplements, cardiometabolic markers of health, and variants in the FADS1/2 gene cluster. Nevertheless, findings from the present work should be expanded in future studies to continue this line of investigation. For example, future studies could include a placebo control group to account for any extraneous variables potentially contributing to changes in the various study endpoints, and a food frequency questionnaire could be provided to participants in order to more accurately monitor dietary habits. In addition, RNA could be collected to examine the effect of fish oil supplements on FADS1 and FADS2 gene expression.

## 5. Conclusions

Our study demonstrated that fish oil providing 1.8 g EPA/DHA per day caused a reduction in circulating levels of TAG and glucose, and significantly altered levels of circulating FAs in young healthy men. Importantly, normalization of TAG and glucose following the washout period reinforces the necessity of continued supplementation in order to maintain these reductions in cardiometabolic markers. Further, studying dynamic changes in FA profiles during the 20-week study period showed that EPA and DHA enrichment persists for at least eight weeks in the RBC fraction following supplementation; reinforcing the importance of a washout period of sufficient time in future clinical trials. We have also shown that genotype may be a potential mediator of an individual’s response to fish oil supplementation, most notably with regards to EPA levels. Overall, our study has demonstrated that young adults provided with a moderate daily dose of fish oil supplements experience reductions in some cardiometabolic markers. This information about effects of *n*-3 supplements on cardiometabolic markers, and knowledge of an individual’s FADS1/2 genotype, may help guide the development of personalized strategies to improve long-term health. 

## References

[1] Cordain L., Eaton S.B., Sebastian A., Mann N., Lindeberg S., Watkins B.A., O’Keefe J.H., Brand-Miller J. (2005). Origins and evolution of the western diet: Health implications for the 21st century. Am. J. Clin. Nutr..

[2] Cleland L.G., James M.J., Proudman S.M. (2006). Fish oil: What the prescriber needs to know. Arthritis Res. Ther..

[3] Kris-Etherton P.M., Harris W.S., Appel L.J. (2003). Fish consumption, fish oil, omega-3 fatty acids, and cardiovascular disease. Arterioscler. Thromb. Vasc. Biol..

[4] Tur J., Bibiloni M., Sureda A., Pons A. (2012). Dietary sources of omega 3 fatty acids: Public health risks and benefits. Br. J. Nutr..

[5] Torrejon C., Jung U., Deckelbaum R. (2007). *n*-3 Fatty acids and cardiovascular disease: Actions and molecular mechanisms. Prostaglandins Leukot. Essent. Fatty Acids.

[6] Kromhout D., de Goede J. (2014). Update on cardiometabolic health effects of ω-3 fatty acids. Curr. Opin. Lipidol..

[7] Lorente-Cebrián S., Costa A.G., Navas-Carretero S., Zabala M., Martínez J.A., Moreno-Aliaga M.J. (2013). Role of omega-3 fatty acids in obesity, metabolic syndrome, and cardiovascular diseases: A review of the evidence. J. Physiol. Biochem..

[8] Balk E.M., Lichtenstein A.H., Chung M., Kupelnick B., Chew P., Lau J. (2006). Effects of omega-3 fatty acids on serum markers of cardiovascular disease risk: A systematic review. Atherosclerosis.

[9] Eslick G.D., Howe P.R., Smith C., Priest R., Bensoussan A. (2009). Benefits of fish oil supplementation in hyperlipidemia: A systematic review and meta-analysis. Int. J. Cardiol..

[10] Hartweg J., Perera R., Montori V., Dinneen S., Neil H., Farmer A.  (2008). Omega-3 polyunsaturated fatty acids (PUFA) for type 2 diabetes mellitus. Cochrane Database Syst. Rev..

[11] Karaouzene N., Merzouk H., Aribi M., Merzouk S., Yahia Berrouiguet A., Tessier C., Narce M. (2011). Effects of the association of aging and obesity on lipids, lipoproteins and oxidative stress biomarkers: A comparison of older with young men. Nutr. Metab. Cardiovasc. Dis..

[12] Janssen I. (2009). Influence of age on the relation between waist circumference and cardiometabolic risk markers. Nutr. Metab. Cardiovasc. Dis..

[13] Kitson A.P., Stroud C.K., Stark K.D. (2010). Elevated production of docosahexaenoic acid in females: Potential molecular mechanisms. Lipids.

[14] Hodson L., Skeaff C.M., Fielding B.A. (2008). Fatty acid composition of adipose tissue and blood in humans and its use as a biomarker of dietary intake. Prog. Lipid Res..

[15] Metherel A., Armstrong J., Patterson A., Stark K. (2009). Assessment of blood measures of *n*-3 polyunsaturated fatty acids with acute fish oil supplementation and washout in men and women. Prostaglandins Leukot. Essent. Fatty Acids.

[16] Marangoni F., Angeli M.T., Colli S., Eligini S., Tremoli E., Sirtori C.R., Galli C. (1993). Changes of *n*-3 and *n*-6 fatty acids in plasma and circulating cells of normal subjects, after prolonged administration of 20:5 (EPA) and 22:6 (DHA) ethyl esters and prolonged washout. Biochem. Biophys. Acta.

[17] Katan M., Deslypere J., van Birgelen A., Penders M., Zegwaard M. (1997). Kinetics of the incorporation of dietary fatty acids into serum cholesteryl esters, erythrocyte membranes, and adipose tissue: An 18-month controlled study. J. Lipid Res..

[18] Cao J., Schwichtenberg K.A., Hanson N.Q., Tsai M.Y. (2006). Incorporation and clearance of omega-3 fatty acids in erythrocyte membranes and plasma phospholipids. Clin. Chem..

[19] Merino D.M., Ma D., Mutch D.M. (2010). Genetic variation in lipid desaturases and its impact on the development of human disease. Lipids Health Dis..

[20] Kwak J.H., Paik J.K., Kim O.Y., Jang Y., Lee S.-H., Ordovas J.M., Lee J.H. (2011). FADS gene polymorphisms in Koreans: Association with ω-6 polyunsaturated fatty acids in serum phospholipids, lipid peroxides, and coronary artery disease. Atherosclerosis.

[21] Kröger J., Schulze M.B. (2012). Recent insights into the relation of δ5 desaturase and δ6 desaturase activity to the development of type 2 diabetes. Curr. Opin. Lipidol..

[22] Simopoulos A.P. (2010). Genetic variants in the metabolism of omega-6 and omega-3 fatty acids: Their role in the determination of nutritional requirements and chronic disease risk. Exp. Biol. Med..

[23] Lattka E., Illig T., Heinrich J., Koletzko B. (2010). Do fads genotypes enhance our knowledge about fatty acid related phenotypes?. Clin. Nutr..

[24] Roke K., Ralston J.C., Abdelmagid S., Nielsen D.E., Badawi A., El-Sohemy A., Ma D.W., Mutch D.M. (2013). Variation in the fads1/2 gene cluster alters plasma *n*-6 pufa and is weakly associated with hscrp levels in healthy young adults. Prostaglandins Leukot Essent. Fatty Acids.

[25] Martinelli N., Girelli D., Malerba G., Guarini P., Illig T., Trabetti E., Sandri M., Friso S., Pizzolo F., Schaeffer L. (2008). FADS genotypes and desaturase activity estimated by the ratio of arachidonic acid to linoleic acid are associated with inflammation and coronary artery disease. Am. J. Clin. Nutr..

[26] Cormier H., Rudkowska I., Paradis A.-M., Thifault E., Garneau V., Lemieux S., Couture P., Vohl M.-C. (2012). Association between polymorphisms in the fatty acid desaturase gene cluster and the plasma triacylglycerol response to an *n*-3 PUFA supplementation. Nutrients.

[27] Gillingham L.G., Harding S.V., Rideout T.C., Yurkova N., Cunnane S.C., Eck P.K., Jones P.J. (2013). Dietary oils and FADS1-FADS2 genetic variants modulate [13c] α-linolenic acid metabolism and plasma fatty acid composition. Am. J. Clin. Nutr..

[28] Al-Hilal M., AlSaleh A., Maniou Z., Lewis F.J., Hall W.L., Sanders T.A., O’Dell S.D. (2013). Genetic variation at the FADS1-FADS2 gene locus influences delta-5 desaturase activity and LC-PUFA proportions after fish oil supplement. J. Lipid Res..

[29] Lawson L.D., Hughes B.G. (1988). Absorption of eicosapentaenoic acid and docosahexaenoic acid from fish oil triacylglycerols or fish oil ethyl esters co-ingested with a high-fat meal. Biochem. Biophys. Res. Commun..

[30] Merino D.M., Johnston H., Clarke S., Roke K., Nielsen D., Badawi A., El-Sohemy A., Ma D.W., Mutch D.M. (2011). Polymorphisms in FADS1 and FADS2 alter desaturase activity in young Caucasian and Asian adults. Mol. Genet. Metab..

[31] dbSNP Short Genetic Variations. http://www.ncbi.nlm.nih.gov/SNP/.

[32] Lattka E., Illig T., Koletzko B., Heinrich J. (2010). Genetic variants of the FADS1 FADS2 gene cluster as related to essential fatty acid metabolism. Curr. Opin. Lipidol..

[33] Tanaka T., Shen J., Abecasis G.R., Kisialiou A., Ordovas J.M., Guralnik J.M., Singleton A., Bandinelli S., Cherubini A., Arnett D. (2009). Genome-wide association study of plasma polyunsaturated fatty acids in the inchianti study. PLoS Genet..

[34] SNAP SNP Annotation and Proxy Search. http://www.broadinstitute.org/mpg/snap.

[35] Pettersson F.H., Anderson C.A., Clarke G.M., Barrett J.C., Cardon L.R., Morris A.P., Zondervan K.T. (2009). Marker selection for genetic case-control association studies. Nat. Protoc..

[36] Holub B., Mutch D.M., Pierce G.N., Rodriguez-Leyva D., Aliani M., Innis S., Yan W., Lamarche B., Couture P., Ma D.W. (2014). Proceedings from the 2013 canadian nutrition society conference on advances in dietary fats and nutrition. Appl. Physiol. Nutr. Metab..

[37] Leiter L.A., Fitchett D.H., Gilbert R.E., Gupta M., Mancini G., McFarlane P.A., Ross R., Teoh H., Verma S., Anand S. (2011). Cardiometabolic risk in canada: A detailed analysis and position paper by the cardiometabolic risk working group. Can. J. Cardiol..

[38] Pereira M.A., Jacobs D.R., Pins J.J., Raatz S.K., Gross M.D., Slavin J.L., Seaquist E.R. (2002). Effect of whole grains on insulin sensitivity in overweight hyperinsulinemic adults. Am. J. Clin. Nutr..

[39] Harris W.S., Pottala J.V., Sands S.A., Jones P.G. (2007). Comparison of the effects of fish and fish-oil capsules on the *n*-3 fatty acid content of blood cells and plasma phospholipids. Am. J. Clin. Nutr..

[40] Von Schacky C., Fischer S., Weber P.C. (1985). Long-term effects of dietary marine omega-3 fatty acids upon plasma and cellular lipids, platelet function, and eicosanoid formation in humans. J. Clin. Investig..

[41] Adkins Y., Kelley D.S. (2010). Mechanisms underlying the cardioprotective effects of omega-3 polyunsaturated fatty acids. J. Nutr. Biochem..

[42] Pinel A., Morio-Liondore B., Capel F. (2013). *n*-3 Polyunsaturated fatty acids modulate metabolism of insulin-sensitive tissues: Implication for the prevention of type 2 diabetes. J. Physiol. Biochem..

[43] Zulyniak M.A., Perreault M., Gerling C., Spriet L.L., Mutch D.M. (2013). Fish oil supplementation alters circulating eicosanoid concentrations in young healthy men. Metabolism.

[44] Fekete K., Marosvölgyi T., Jakobik V., Decsi T. (2009). Methods of assessment of *n*-3 long-chain polyunsaturated fatty acid status in humans: A systematic review. Am. J. Clin. Nutr..

[45] Barceló-Coblijn G., Murphy E.J., Othman R., Moghadasian M.H., Kashour T., Friel J.K. (2008). Flaxseed oil and fish-oil capsule consumption alters human red blood cell *n*-3 fatty acid composition: A multiple-dosing trial comparing 2 sources of *n*-3 fatty acid. Am. J. Clin. Nutr..

[46] Willer C.J., Schmidt E.M., Sengupta S., Peloso G.M., Gustafsson S., Kanoni S., Ganna A., Chen J., Buchkovich M.L., Global Lipids Genetics Consortium (2013). Discovery and refinement of loci associated with lipid levels. Nat. Genet..

[47] Schaeffer L., Gohlke H., Müller M., Heid I.M., Palmer L.J., Kompauer I., Demmelmair H., Illig T., Koletzko B., Heinrich J. (2006). Common genetic variants of the FADS1 FADS2 gene cluster and their reconstructed haplotypes are associated with the fatty acid composition in phospholipids. Hum. Mol. Genet..

[48] Keenan A.H., Pedersen T.L., Fillaus K., Larson M.K., Shearer G.C., Newman J.W. (2012). Basal omega-3 fatty acid status affects fatty acid and oxylipin responses to high-dose *n*3-hufa in healthy volunteers. J. Lipid Res..

